# Too Big or Too Small? The Threshold Effects of City Size on Regional Pollution in China

**DOI:** 10.3390/ijerph19042184

**Published:** 2022-02-15

**Authors:** Xiong Chen, Wencui Du

**Affiliations:** 1 College of Humanities and Social Sciences, Nanjing University of Aeronautics and Astronautics, Nanjing 211106, China; chenxiong@china-ceco.com; 2 School of Economics, Capital University of Economics and Business, Beijing 100070, China

**Keywords:** city size, environmental pollution, threshold effect model

## Abstract

The relationship between urban agglomeration and environmental pollution was checked using the balanced panel data of 285 cities in China from 2003 to 2016 and applying the fixed-effect model and the threshold effect model. This showed that: (1) the relationship between urban agglomeration (represented by city size) and environmental pollution is not linear but an inverted U-shape. As long as the GDP is less than 800,370 million RMB, the expansion of city size is not conducive to reducing pollutant emissions. When GDP is less than 41,641 million RMB, the influence of city expansion on environmental pollution is relatively less. When GDP is higher than 800,370 million RMB, the city expansion may reduce pollutant emission. (2) The city size is not too big but is in fact too small. Only 18 cities experienced the inverted U-shape with the expansion of their city size, causing the gas and water pollutant emissions to decrease. (3) For cities in an urban agglomeration, environmental pollution can be reduced by expanding the city size through coordinated development of urban agglomeration. In conclusion, for most large cities in urban agglomerations in China, the city size is not too large but too small.

## 1. Introduction

The history of city development stems from the history of the migration from the rural area to the urban area and a history of industrial agglomeration. Cities play a critical role in the development of a country, as they are the gathering places of a country’s main economy and the living space for much of the population. Cities are also the growth poles of economic development. With the advancement of industrialization and urbanization, city sizes continue to expand gradually, and megacities are continually emerging, therefore making the relationship between urban development and economic development inseparable.

**Figure 1:**
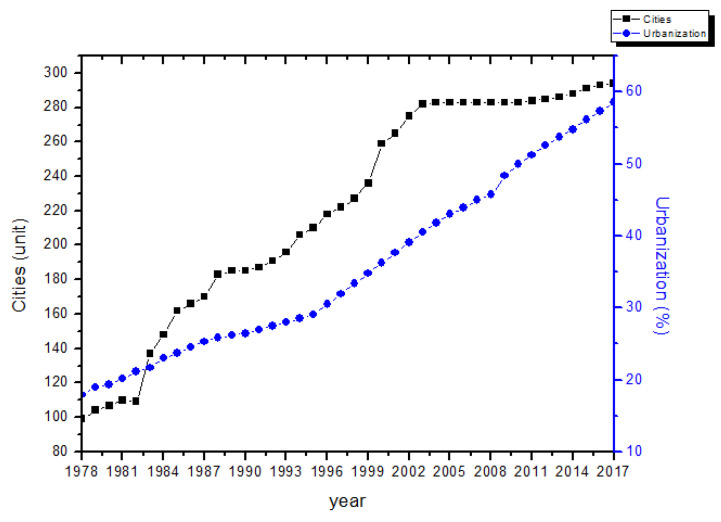
Divisions of administrative areas and urbanization rate in China (1978–2017). Sources: China Compendium of Statistics (1949–2008), China Statistical Yearbook (2010–2018).

In the case of China, since the reform and opening-up in 1978, China has entered the rapid urbanization stage, with a rapid increase in the number of cities and the urban population. Figure 1 depicts the historical changes in the urbanization rate and prefecture-level cities in China from 1978 to 2017. Between 1978 and 2017, China was the largest developing country in the world, and there was a great migration in the population from rural to urban areas. In 1978, the population living in cities and towns was 172.45 million. This had increased more than 4.7 times by 2017 to a population of 813.47 million living in cities and towns. Approximately 52 million people migrated from the countryside to the city each year, excluding individuals who already worked in the city. Notably, household registration remains, resulting in the existence of a registered rural population living in cities. Considering these people, China’s urbanization rate increased dramatically.

The “space-time compression” effect is a consequence of the rapid urban development in China. This means that some big cities have completed the urbanization process of developed countries in a relatively short period. However, the continuous expansion of cities has gradually exposed a number of problems. The excessive agglomeration of the population, industry, and transportation to cities, in combination with unscientific urban planning, has meant that urban expansions exceed the affordability of social resources. This has resulted in a series of economic and social problems, which have mainly manifested in population expansion, resource shortage, environmental deterioration, housing shortage, and traffic congestion. Environmental degradation is one of the economic and social problems caused by the expansion of city size and is particularly prominent in developing countries.

For example, when looking at urban air pollution in China, in 2017, 239 out of 338 cities exceeded the limits for air with acceptable quality (including municipalities directly under the Central Government, prefecture-level cities, autonomous prefectures, and leagues), accounting for 70.7% of all cities. Meanwhile, only 99 cities complied with the environmental air quality standards, accounting for the other 29.3%. The average number of days exceeding the standard in 338 cities was 22.0%, meaning there were 2311 days of severe pollution and 802 days of serious pollution, and 48 cities had more than 20 days of severe pollution.

Furthermore, some people attribute “city diseases”, such as environmental pollution, to the rapid growth of the urban population and city size expansion. The question to be addressed is whether environmental pollution in big cities is caused by the increasing size of the city. Researchers observe air and water pollution in big cities, and there is a common belief that a larger population causes more pollution. However, the researchers assert that it may only because the pollution of big cities is of greater concern. If city size is a critical factor affecting environmental pollution, what city size is optimal for environmental protection? From the perspective of sustainable development, is there an optimal city size? These questions are worthy of further study. However, according to our literature review, there is currently no agreement of the answers to these questions.

The theoretical discussion of optimal city size in the literature began with Henderson [1], who presented a general equilibrium model of an economy where production and consumption occurred in cities and argued that the city size equilibrium is determined by the location or investment decisions of laborers and capital owners. Consequently, a series of studies expanded on this theory from different perspectives and discussed, in-depth, what the optimal city size is, and whether a city can be too big or too small [2,3,4]. Moreover, studies have explored the impact of city size on employment [5,6,7] and on technological progress [8].

This paper aims to make two main contributions. Notably, in the literature review, the linear relationship between city size and environmental pollution has been assessed, but no decisive conclusion was reached. Thus, the first contribution of this paper is to test the non-linear relationship. The second contribution is that policy suggestions are proposed according to the inflection points of the inverted U-shape.

Therefore, this paper is structured as follows. In Section 2, we present our regression model and describe the data. The empirical results are discussed in Section 3, and further analyses about the inflection point between city size and environmental pollution are presented in Section 4, followed by the conclusions in Section 5 and suggestions in Section 6.

## 2. Literature Review

Research on the relationship between city size and environmental pollution has been related to two research strands. One strand uses mathematical models to explore the optimal city size in theory. Whilst the other focuses more on the historical data and uses empirical research to find the appropriate relationship between city size and environmental pollution.

Regarding the theoretical literature, the effect of pollution on city size equilibrium was not debated until Borck and Tabuchi [9]. Borck and Tabuchi [9] studied the optimal size and the equilibrium size of cities in a monocentric city model with environmental pollution based on Henderson [1]. They considered that if pollution is local, the equilibrium cities are too large. If pollution is global and per capita pollution declines with city size, the equilibrium cities may be too small. Based on Henderson [1], Pflüger and Michael [10] developed a micro-founded city systems model with an endogenous number of cities to explore whether local governments establish optimal city size when production processes involve environmental pollution. The belief was that if pollution is local, the equilibrium cities are at the optimal size, and if pollution is global, the cities are too small. This conclusion is in opposition to the findings of Borck and Tabuchi [9].

Compared to the less theoretical studies, there are many empirical studies on city size and environmental pollution, and these studies can be split into two main groups based on their conclusions. The first group considers that the expansion of city size will enable large cities to have better pollution control equipment and stronger environmental governance capacity than smaller cities, therefore helping to alleviate environmental pollution [11,12,13,14,15].

Satterthwaite [11] studied urban environmental problems in developing countries in Asia, Africa, and Latin America and found that because of the more advanced pollution control technology in big cities, as well as the higher pressure on them and their ability to conduct environmental governance, environmental pollution would be mitigated with the expansion of city size and that the environmental problems in smaller cities were the most serious. Kahn [12] considered the larger metropolitan areas as the primary income growth locations, especially in developing countries, as these areas have more resources and more educated residents, both of which lead to a reduction in pollution levels. Glaeser [13] believed that less gasoline is consumed as more people commute using public transport and houses are smaller. All these factors mean that cities are, in fact, green, in the sense that they use less energy and have smaller carbon footprints than less dense living arrangements. Moreover, Gaigné et al. [14] argued that a higher population density makes cities more environmentally friendly because the average commuting length is reduced. In addition, Kahn and Walsh [15] believed that big cities feature more high-tech companies instead of heavy industries and employ workers who demand high-quality local amenities.

The second strand of research considers that the expansion of city size aggravates the consumption of resources and environmental pollution [16,17]. Li et al. [16] explored the impact of city size change and industrial structure change on CO_2_ emissions using 50 Chinese cities of different sizes from 2005 to 2014. They found that increasing city size increases CO_2_ emissions and the impacts on CO_2_ emissions in different sized cities can be significant. Liu et al. [17] investigated how urban forms were related to different air pollution measures (PM_2.5_ and API) in 83 Chinese cities and found that urban air pollution levels increased consistently and substantially from small to medium to large and finally to megacities.

According to both the theoretical and empirical studies, no unified conclusion has been reached regarding the relationship between city size and environmental pollution. Thus, with the expansion of city sizes, will environmental pollution increase in severity or gradually be alleviated? Why do empirical tests show both a negative and a positive correlation between city size and environmental pollution?

The non-linear relationship between city size and environmental pollution is tested in this study for the reasons mentioned above. Environmental pollution was measured using industrial wastewater, industrial SO_2_, industrial dust discharge, and PM_2.5_. To measure city size and environmental pollution from different aspects, we represent the city size using the population, gross domestic product (GDP), and built-up area. According to the “Notice on Adjusting the Standards for Classifying Cities” issued by The State Council in China, cities are classified according to population, and big cities usually have a larger built-up area and higher GDP level. In order to fully measure the city size, we not only use the population indicators in the empirical test, but also supplement the regression results from the perspectives of GDP and built-up area.

## 3. Methodology and Data

### 3.1. Empirical Research Design

#### 3.1.1. Basic Model

To investigate the impact of city size on environmental pollution and whether there is a nonlinear effect, the empirical model was established based on the STIRPAT model, which is a widely used model to investigate the level of environmental pollution and greenhouse gas emissions. The STIRPAT model is based on the influence, population, affluence, and technology (IPAT) model proposed by Ehrlich and Holdren [18]. The econometric model is established as follows:

(1) $$\begin{array}{cc} \ln\left(POLLU_{it}\right) & =\alpha_{0}+\alpha_{1}\ln\left(SIZE_{it}\right)+\alpha_{2}\ln{\left(SIZE_{it}\right)}^{2}+\alpha_{3}\ln\left(IND_{it}\right)+\alpha_{4}\ln\left(FDI_{it}\right)\\ & +\alpha_{5}\ln\left(TECH_{it}\right)+\alpha_{6}\ln\left(ENERGY_{it}\right)+\alpha_{7}\ln\left(INVEST_{it}\right)+\mu_{i}+\tau_{t}+\varepsilon_{it} \end{array}$$

where the dependent variable is POLLU, which represents the total pollutant emissions. In the regression, the pollutant emissions are measured using three indicators: industrial wastewater discharge, industrial SO_2_ discharge, and industrial dust discharge. The unit of industrial wastewater discharge is a million tons, the unit of industrial SO_2_ discharges is a thousand tons, and the unit of industrial dust discharge is also a thousand tons.

The independent variable is SIZE, which represents the city size. Using the concepts of physics, Way [19] considered that city size could be measured from three aspects: the area, the population, and the volume. In accordance with this, we measured city size from three perspectives: population, GDP, and built-up area. The unit of population is ten thousand, the unit of GDP is one billion RMB, and the unit of area is a square kilometer.

To investigate whether the city size has a nonlinear impact on environmental pollution, the quadratic term of SIZE was added to the empirical model. From the regression results, if $\hat{\alpha_{1}}>0$, the larger the city size, the more pollution emissions, contrastingly if $\hat{\alpha_{1}}<0$, the larger the city size, the fewer pollution emissions. If $\hat{\alpha_{1}}>0$, but $\hat{\alpha_{2}}<0$, the relationship between city size and environmental pollution is nonlinear. With the expansion of city size, environmental pollution presents an inverted U-shaped relationship, which first increases and decreases at an inflection point. If $\hat{\alpha_{1}}<0$, but $\hat{\alpha_{2}}>0$, the nonlinear relationship between city size and environmental pollution can also be proven. With the expansion of city size, environmental pollution presents a U-shaped relationship, decreasing and then increasing. If $\hat{\alpha_{1}}$ and $\hat{\alpha_{2}}$ are identical, or $\hat{\alpha_{2}}$ is not significant, the relationship between city size and environmental pollution is linear.

The control variables include *IND*, *FDI*, *TECH*, *ENERGY*, and *INVEST*. *IND* refers to the proportion of industry value added to GDP, measured in units of 1%. The higher the proportion of industrial value added to GDP, the more pollution emissions from industrial production. *FDI* refers to foreign direct investment (FDI), measured in units of billion dollars. The research on the relationship between FDI and environmental pollution has not been uniform, with some studies asserting FDI aggravates environmental pollution, that is, the “Pollution Haven Hypothesis” [20]. *TECH* refers to the proportion of scientific expenditure in the general budget expenditure of local finance, measured in units of 1%. According to the literature, technological progress may reduce resource consumption per unit output, reduce pollution emissions [21], or increase total output and pollution emissions [22]. *ENERGY* refers to annual electricity consumption, measured in units of billion-kilowatt hour. Energy use is closely related to the pollutant emissions in urbanization [23,24]; thus, energy consumption was added as a control variable. The annual electricity consumption data was selected instead of the energy consumption data based on data availability. *INVEST* refers to the total fixed assets investment, measured in units of a billion RMB. Fixed assets investment is a critical factor affecting economic growth and one of the factors causing environmental pollution. Therefore, *INVEST* has been added to the empirical model to control the influence of fixed asset investments.

Additionally, *I* = 1…n identifies the city, t refers to the yearly observation, *µ* represents the individual factors of the city that are not affected by time, *τ* represents the time factors that are not affected by the city’s individual factors and *ε* is the error term.

#### 3.1.2. Threshold Model

The threshold model, introduced by Hansen [25], describes the jumping character or structural break in the relationship between variables. Hansen [25] provided the least squares estimation method for threshold fixed effect regression. Then, the threshold regression for panel data was complemented by Wang [26]. Considering the single-threshold model, the equation is

(2) $$y_{it}=\left\{ \begin{array}{l} \mu_{i}+\beta_{1}^{\prime }x_{it}+\varepsilon_{it},\\ \mu_{i}+\beta_{2}^{\prime }x_{it}+\varepsilon_{it}, \end{array}\right.\begin{array}{l} if\\ if \end{array}\begin{array}{l} q_{it}\leq \gamma \\ q_{it}>\gamma \end{array}$$

*y_it_* is the dependent variable, *x_it_* is the independent variable, *q_it_* is the threshold variable, and γ is the threshold. Here, the observations are divided into two regimes, with coefficients *β*_1_ and *β*_2_ depending on whether the threshold variable *q_it_* is either smaller or larger than the threshold γ. Their differing regression slopes distinguish the regimes.

Using the indicator function *I*(·), a simple alternative of Equation (2) is

(3) $$y_{it}=\mu_{i}+\beta_{1}^{\prime }x_{it}\cdot I\left(q_{it}\leq \gamma \right)+\beta_{2}^{\prime }x_{it}\cdot I\left(q_{it}>\gamma \right)+\varepsilon_{it}$$

Defining $\beta =\left(\begin{array}{l} \beta_{1}\\ \beta_{2} \end{array}\right)$ and $x_{it}\left(\gamma \right)=\left(\begin{array}{l} x_{it}\cdot I\left(q_{it}\leq \gamma \right)\\ x_{it}\cdot I\left(q_{it}>\gamma \right) \end{array}\right)$, Equation (3) can be further simplified as follows:

(4) $$y_{it}=\mu_{i}+\beta^{\prime }x_{it}\left(\gamma \right)+\varepsilon_{it}$$

By averaging the time on both sides of Equation (4) simultaneously, Equation (4) can be rewritten as follows:

(5) $${\bar{y}}_{i}=\mu_{i}+\beta^{\prime }{\bar{x}}_{i}\left(\gamma \right)+{\bar{\varepsilon }}_{i}$$

Subtracting Equation (5) from Equation (4), the deviation form of the equation is

(6) $$y_{it}^{*}=\mu_{i}+\beta^{\prime }x_{it}^{*}\left(\gamma \right)+\varepsilon_{it}^{*}$$

where $y_{it}^{*}\equiv y_{it}-{\bar{y}}_{i}$, $x_{it}^{*}\left(\gamma \right)\equiv x_{it}\left(\gamma \right)-{\bar{x}}_{i}\left(\gamma \right)$, and $\varepsilon_{it}^{*}\equiv \varepsilon_{it}-{\bar{\varepsilon }}_{i}$. Using the two-step regression method, first, given γ, the ordinary least-squares estimator of *β* is

(7) $$\hat{\beta }\left(\gamma \right)={\left\{x^{*}{\left(\gamma \right)}^{\prime }x^{*}\left(\gamma \right)\right\}}^{-1}\left\{x^{*}{\left(\gamma \right)}^{\prime }y^{*}\right\}$$

Second, $\hat{\gamma }$ is the value that minimizes the residual sum of squares (SSR). Finally, the estimated coefficient is $\hat{\beta }\left(\hat{\gamma }\right)$.

Next, we assess whether *H*_0_: $\beta_{1}=\beta_{2}$. Hansen [25] proposed using the likelihood ratio test (LR):

(8) $$LR\left(\gamma \right)\equiv \left[SSR^{*}-SSR\left(\hat{\gamma }\right)\right]/{\hat{\sigma }}^{2}$$

where ${\hat{\sigma }}^{2}\equiv \frac{SSR\left(\hat{\gamma }\right)}{n\left(T-1\right)}$. If “*H*_0_: $\beta_{1}=\beta_{2}$” is rejected, the threshold effect is considered, and the threshold value can be further tested. Defining LR statistics:

(9) $$LR\left(\gamma \right)\equiv \left[SSR\left(\gamma \right)-SSR\left(\hat{\gamma }\right)\right]/{\hat{\sigma }}^{2}$$

Here, we identify the threshold effect using a bootstrap method designed by Hansen [25]. Above is the estimated scheme search for any single threshold. The method for searching double or triple thresholds is similar.

To investigate the threshold effect of city size on pollutant emissions, the econometric model is as follows:

(10) $$\begin{array}{cc} \ln\left(POLLU_{it}\right) & =\beta_{0}+\beta_{1}I\left(SIZE_{it}\leq \gamma \right)\ln\left(SIZE_{it}\right)+\beta_{2}I\left(SIZE_{it}>\gamma \right)\ln\left(SIZE_{it}\right)\\ & +\beta_{3}\ln\left(IND_{it}\right)+\beta_{4}\ln\left(FDI_{it}\right)+\beta_{5}\ln\left(TECH_{it}\right)\\ & +\beta_{6}\ln\left(ENERGY_{it}\right)+\beta_{7}\ln\left(INVEST_{it}\right)+\mu_{i}+\tau_{t}+\varepsilon_{it} \end{array}$$

where the variable definitions are similar to Equation (1) (Section 3.1).

### 3.2. Sample Selection

Our objective was to identify the relationship between city size and pollutant emissions. A cross-city panel data set was selected. The data for all variables were obtained from the China City Statistical Yearbook (2004–2017). The sample list, which included 285 Chinese cities from 2003 to 2016, and the total observations were 3990.

From 2003 to 2016, the number of cities at prefecture level and above in China was constantly changing. To obtain balanced panel data, we made fine adjustments to the sample cities: (1) Because of the lack of data, Lhasa was deleted. (2) In 2013, Chaohu was merged into Hefei, Wuhu, and Ma’anshan; thus, the data after 2013 were missing, and Chaohu was deleted. (3) In 2013, four new prefecture level cities—Sansha, Bijie, Tongren, and Haidong—were established in China. Due to the lack of data before 2013, the four cities were deleted. (4) In 2015, Danzhou was established. Due to the lack of data before 2015, Danzhou was deleted.

### 3.3. Summary Statistics

The summary statistics of the key variables are presented in Table 1. In Table 1, the mean of the variable *population* is 431.85. This result indicates that the average population in a Chinese city is 431.85 ten thousand. The maximum population is 3392 ten thousand (Chongqing in 2016). The minimum value is 16.37 ten thousand (Jiayuguan in 2003). It shows the great differences in the size of Chinese cities. The maximum is 207 times the minimum. In terms of GDP, the maximum value of *gdp* is 28178.650 billion RMB (Shanghai in 2016), and the minimum value is 31.773 billion RMB (Jiayuguan in 2003). From the urban land area perspective, the maximum value of *area* is 1420 square kilometers (Beijing in 2016), and the minimum value is 5 square kilometers (Dingxi in 2003). From the perspective of population, economic development, and urban land area, great differences are observed in city size and development in Chinese cities. The scale gap between big cities and small cities is large.

## 4. Results

### 4.1. Basic Regression Results

The regression results are presented in Table 2. The fixed effect regressions using industrial wastewater to represent environmental pollution are presented in Column (1). The regressions using industrial SO_2_ to represent environmental pollution are presented in Column (2). The regressions using industrial dust to represent environmental pollution are presented in Column (3).

In Table 2, Column (1), (2), and (3), *SIZE* has a positive and significant coefficient, suggesting the positive relationship between population and environmental pollution, while in Column (1), (2), and (3), *SIZE*^2^ has a negative and significant coefficient. The coefficients of *SIZE* and *SIZE*^2^ together prove that the relationship between environmental pollution and city size represented by population is an inverted U-shape, suggesting that when the city’s population increases gradually, the pollution emissions first increase and then decrease after the inflection point.

When the urban population is small, it is suggested that the expansion of city size, industry, and the population is concentrated in urban areas. The city attracts labor force, and the inflow of labor force further promotes the city’s development. Then, the further city development again attracts more people. When the economic development and population inflow in cities show a spiral escalation trend, the city size can be further expanded. However, with the expansion of city size, environmental problems caused by agglomeration still emerge. First, industrial production requires skilled industrial workers, and most industrial workers live in cities. Thus, industrial production must be concentrated in cities during the early stage of economic and social development. Industrial agglomeration results in increased production and consumption of resources and energy. Second, with the interactive development of urbanization and industrialization, the population is constantly gathering in cities, especially in large cities. When people migrate from rural areas to the city, they have changed their household registration status and living habits, resulting in increased consumer demand. Therefore, as the city size expands, the environmental pollution increases and environmental problems become increasingly prominent.

However, the relationship between city size and environmental pollution is not unchanged. When the city size expands to a certain extent, urban development will produce an agglomeration economy.

On the one hand, industrial agglomeration can promote the centralized utilization of resources. The externality of knowledge and technology will accelerate the diffusion, and resource use efficiency will continue to improve. In large cities, the government has more finances, capacity, and energy to promote its environmental regulations and environmental governance actively. Improvement of these institutional factors contributes to the reduction of pollution emissions. On the other hand, population agglomeration also has economies of scale effect. When the city size increases, people will benefit from city development with their living standards gradually improved. When the living demands have been fulfilled, people begin to pay more attention to environmental quality: they will choose more green label products and pay more attention to environmental protection.

People’s awareness of environmental protection will improve through the transmission of knowledge and ideas in their daily life. Additionally, with the expansion of city size, the construction of the environmental infrastructure will gradually improve. The efficiency of these infrastructures will be higher and more effective, conducive to the realization of pollution reduction and sustainable development. Therefore, with the further expansion of city size, environmental pollution will not continue to increase but will experience a downturn, and environmental problems will be alleviated.

In addition, the results of the control variables also have some valuable results. *IND* has a positive and significant coefficient, suggesting that a city with a higher proportion of the secondary industry is more likely to cause more pollutant emissions. *TECH* has a negative and significant coefficient, suggesting that a city with more advanced technology is more likely to cause fewer pollutant emissions. *ENERGY* has a positive and significant coefficient, which means that a higher energy consumption level is associated with more pollutant emissions. *INVEST* has a negative and significant coefficient, suggesting that the fixed assets investment is good for reducing environmental pollution. The only difference is that in Column (5) and (6), environmental pollution is measured using industrial dust, and IND’s estimated coefficient is not significant. The estimated coefficient of *FDI* is significantly negative. Thus, the industrial dust discharge in Chinese cities is related to FDI, and the introduction of FDI is good for pollution control. However, this conclusion is not prominent in the other regressions.

To assess the robustness of the regression results presented in Table 2, we repeated the regression in Table 2 using GDP and built-up area to represent city size instead of population. The regression results are presented in Table 3. The regressions using GDP to represent city size are presented in Column (1), (2), and (3), and the regressions using the built-up area to represent city size are presented in Column (4), (5), and (6). In Table 3, except for the regression in Column (4), all the other regressions are robust. The estimated coefficient of *SIZE* is significantly positive, and the estimated coefficient of *SIZE*^2^ is negative, suggesting that the relationship between city size and environmental pollution is an inverted U-shape. The estimated results of the control variables are almost the same as the results in Table 2.

According to the regression results in Table 2 and Table 3, we calculated the inflection points of an inverted U-shaped relationship. The inflection points calculated are presented in Table 4.

In Table 4, the population’s inflection points are 9.42 million when environmental pollution is represented by industrial wastewater discharge, 2.25 million when environmental pollution is represented by industrial SO_2_ discharge, and 6.09 million when the environmental pollution is represented by industrial dust discharge.

According to the three different inflection points represented by different pollutant emissions, the samples can be divided into four groups. Samples in the first group are 62 cities with a population within 2.25 million, for example, Xiamen, Xining, and Zhuhai. With the expansion of their city size, the three kinds of pollutants gradually increase. Because the urban population is too small to utilize centralized population-based pollution treatment facilities, the scale effect has not yet been achieved. Samples in the second group are 158 cities with a population between 6.09 million and 2.25 million, for example, Dalian, Nanchang, and Taiyuan. With the expansion of their city size, the industrial SO_2_ emissions gradually decrease, but the amount of industrial wastewater and dust continue to increase. The third group samples are 47 cities with a population between 9.42 million and 6.09 million, for example, Nanjing, Suzhou, Hangzhou, and Qingdao. For them, with the expansion of city size, the industrial SO_2_ and dust emissions gradually decrease, but the industrial waste water discharge continues to increase. Thus, for the third group of cities, the gas pollutant emissions are efficiently controlled because of the scale effect. More attention should be paid to the treatment of water pollutant. Samples in the fourth group are 18 cities with a population larger than 9.42 million, including Beijing, Shanghai, and Chongqing, etc. With the expansion of their city size, the gas and water pollutants decrease. Thus, for most of the large cities in China, the city size is not too large but too small. The environmental problems, the so-called “city disease”, are indeed not caused by the expansion of city size but due to the city’s insufficient size.

The inflection points of GDP are 7362 billion RMB when the environmental pollution is represented by industrial wastewater discharge, 68 billion RMB when the environmental pollution is represented by industrial SO_2_ discharge, and 23,461 billion RMB when the environmental pollution is represented by industrial dust discharge. In 2016, the largest GDP appeared in Shanghai and was approximately 2818 billion RMB. Thus, no city achieved the GDP inflection points when environmental pollution is represented by industrial wastewater and dust discharge. For the inflection point of 68 billion RMB, there are only 44 cities with a GDP of less than 68 billion RMB. Thus, with the expansion of most cities’ economic scale, environmental pollution is aggravated in the short term until the inflection point.

The built-up area’ inflection points are 80.40 square kilometers when environmental pollution is represented by industrial SO_2_ and 236.75 square kilometers when environmental pollution is represented by industrial dust. Thus, the sample cities can be divided into three groups.

The first group has 136 cities with their built-up area less than 80.40 square kilometers, for example, Lvliang, Tonghua, and Huangshan. Due to their limitations of a unique geographical location or construction planning, the built-up areas of these cities are not very large, which is not conducive to attracting more people and industries, forming an agglomeration effect, and therefore, the scale effect of pollution reduction cannot be achieved. With the expansion of the built-up area, air pollutants continue to increase. The second group has 105 cities with a built-up area of less than 236.75 square kilometers and more than 80.40 square kilometers, for example, Quanzhou, Weihai, and Jiujiang. For these cities, the enlarging of city size is good for industrial SO_2_ reduction but bad for industrial dust reduction. Only for 44 cities in the third group, including the main large cities of Beijing, Shanghai, and Shenzhen, the expansion of city size reduces air pollutant emission.

### 4.2. Threshold Regression Results

**Figure 2:**
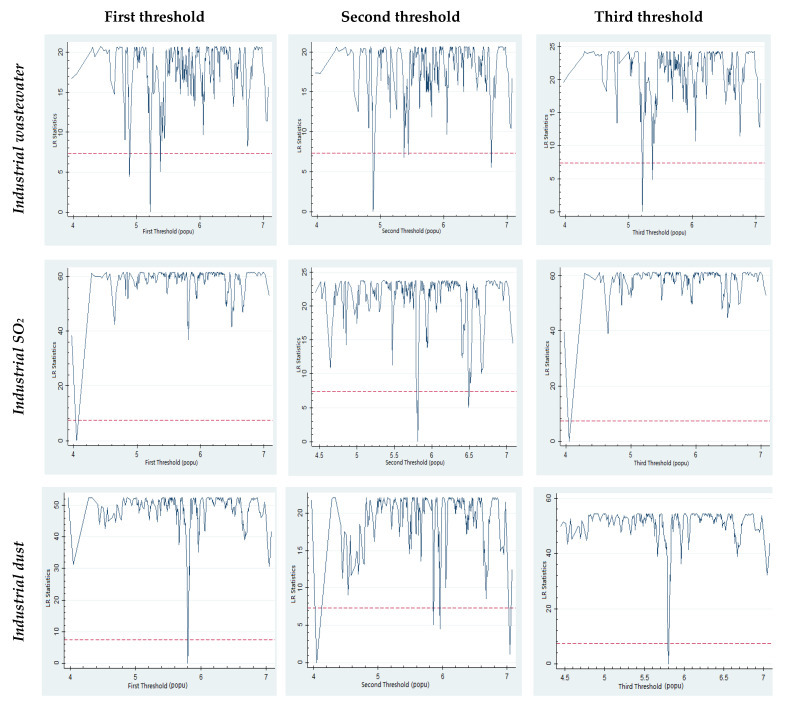
LR test results (*threshold: population*).

Table 5 presents the threshold regression results with *SIZE* represented by population. And the LR test result is presented in Figure 2. As can be seen from Table 5, in the regression where the city size is represented by population, *SIZE* has a positive and significant impact on industrial wastewater only if the population is bigger than 2,160,000. This suggests that if the city population is smaller than 2,160,000, the expansion of city size will not lead to increases in industrial wastewater discharge. However, *SIZE* has a negative and significant impact on industrial SO_2_ discharge when the population is larger than 570,000, indicating that the increasing population may reduce industrial SO_2_ discharge, especially when the population is more than 570,000 and less than 1,040,000. Additionally, *SIZE* has a positive and significant impact on industrial dust discharge when the population is larger than 570,000, suggesting that increases in the population would cause increases in the industrial dust discharge. Therefore, the threshold impact of population on urban environmental pollution is complex. When the population exceeds 570,000, urban size expansion leads to the reduction of industrial SO_2_ emissions but will not reduce industrial dust emissions. The results show that when the population is larger than 570,000, a trade-off should be made for environmental policy between industrial SO_2_ and dust emissions. Additionally, as long as the population is smaller than 2,160,000, the impact of urban expansion on industrial wastewater can be ignored.

**Figure 3:**
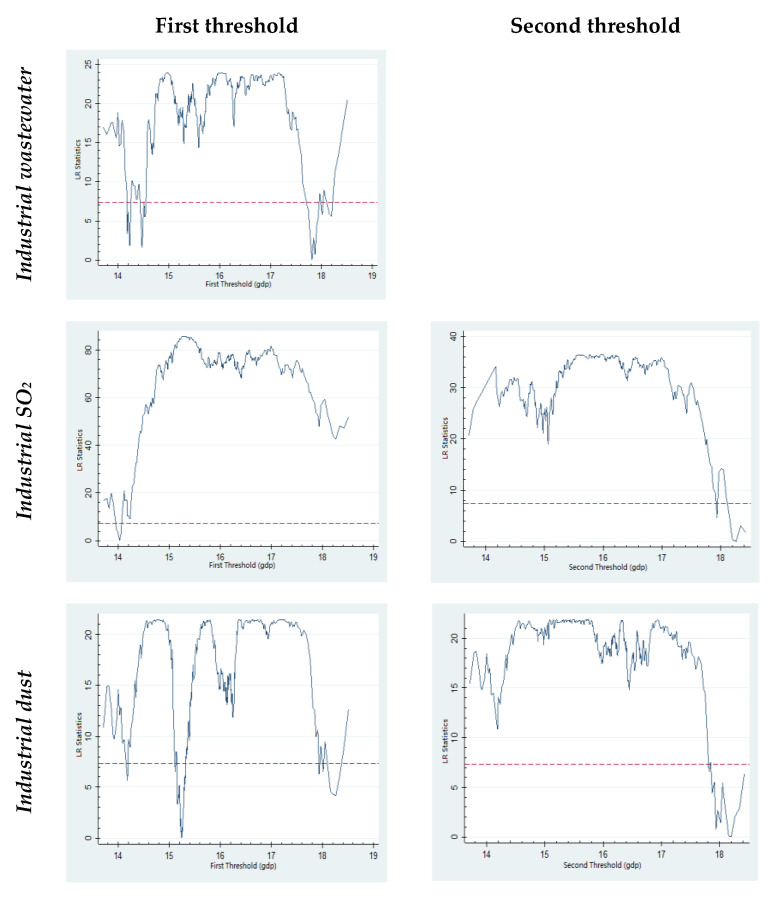
LR test results (*threshold: GDP*).

The threshold regression results with *GDP* represented by population are presented in Table 6, and the LR test result is presented in Figure 3. As presented in Table 6, in the regression where GDP represents the city size, a threshold effect was observed on industrial wastewater discharge, and the threshold is 544,068 million RMB. When the GDP is lower than 544,068 million RMB, the influence of city size on industrial wastewater discharge is not prominent. When the GDP exceeds 544,068 million RMB, the effect of city size on industrial waste water discharge is significantly negative. Additionally, there is a threshold effect with GDP when the city pollutant emissions are represented by industrial SO_2_, and the first and second thresholds are 12,355 million RMB and 850,713 million RMB. When the GDP is less than 12,355 million RMB or more than 850,713 million RMB, the influence of city size on industrial SO_2_ emissions is negative. When the GDP exceeds 12,355 million RMB and is lower than 850,713 million RMB, the effect of city size on industrial SO_2_ is negative, suggesting that the expansion of city size is good for pollutant reduction and the scale effect of city size is prominent.

There is also a threshold effect with GDP if the city pollutant emissions are represented by industrial dust. The first and second thresholds are 41,641 million RMB and 800,370 million RMB. As long as the GDP is less than 800,370 million RMB, the expansion of city size is not conducive to reducing pollutant emissions. However, when GDP is less than 41,641 million RMB, the influence of city expansion on environmental pollution is relatively less. When GDP is higher than 800,370 million RMB, the city expansion increases urban investment for environmental protection, improves pollutant treatment facilities’ efficiency, optimizes resource allocation, and reduces pollutant emission.

**Figure 4:**
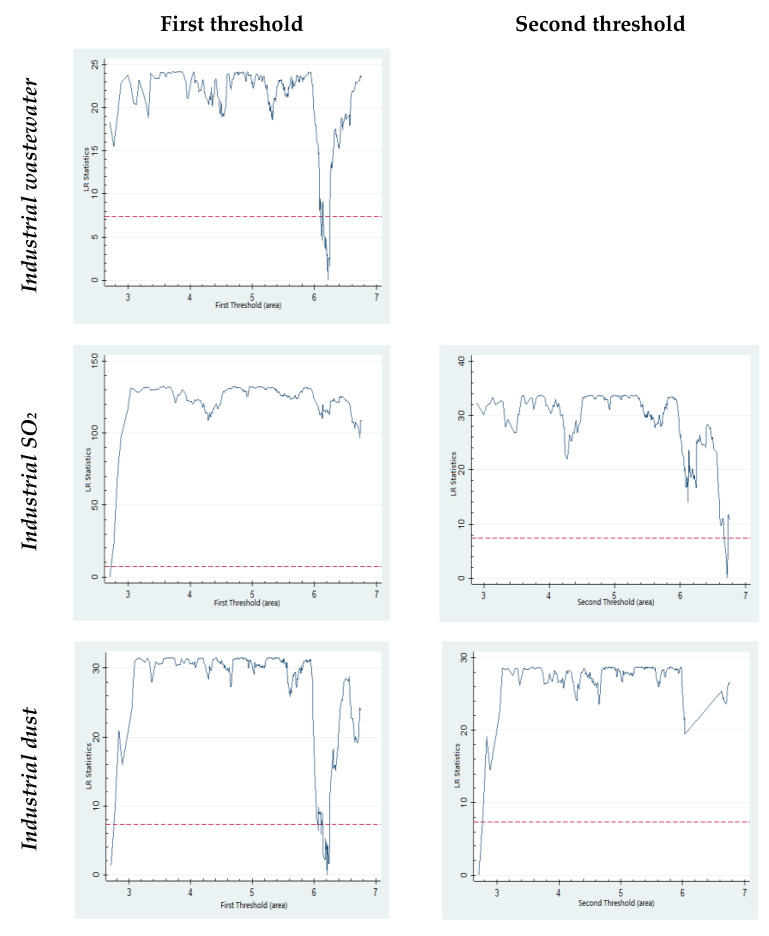
LR test results (*threshold: built-up area*).

The threshold regression results with *GDP* represented by population are presented in Table 7, and the LR test result is presented in Figure 4. As presented in Table 7, in the regression where the built-up area represents the city size, a threshold effect is observed on industrial wastewater discharge. The threshold is 500 square kilometers. If the built-up area is less than 500 square kilometers, the influence of city size on industrial wastewater discharge is positive, suggesting that city expansion harms the environment. However, if the built-up area exceeds 500 square kilometers, the industrial wastewater discharge decreases following the increasing expansion of city size. Thus, the city size is not too large but too small.

Additionally, a threshold effect is observed for the built-up area when the city pollutant emissions are represented by industrial SO_2_. The first and second thresholds are 15 square kilometers and 830 square kilometers, respectively. When the built-up area is smaller than 15 square kilometers or exceeds 830 square kilometers, the expansion of city size is good for industrial SO_2_ reduction. But when the built-up area is bigger than 15 square kilometers and smaller than 830 square kilometers, there is no significant relationship between city size and industrial SO_2_ emissions.

Moreover, the threshold effect of built-up area on industrial dust emissions is significant. When the built-up area is less than 15 square kilometers or exceeds 496 square kilometers, an apparent negative relationship is observed between city size and industrial dust emission, suggesting that the expansion of city size benefits the emission reduction. By contrast, if the built-up area is larger than 15 square kilometers and smaller than 496 square kilometers, the relationship between city size and industrial dust emissions is positive, indicating that the city is too large.

The regression results summary is presented in Table 8. In Table 8, only 54 Chinese cities have a population of less than 2,160,000, such as Fushun in Liaoning, Dongguan in Guangdong, and Dongying Shandong; thus, for most Chinese cities, the population is not too large but too small.

In 2016, the city with the highest GDP was Shanghai with a volume of 2,817,865 million RMB, while the lowest municipal GDP (15,341 Million RMB) occurs in Jiayuguan City. Only 33 cities have a GDP exceeding 544,068 million RMB. Thus, for most cities, the scale effects of GDP have not been achieved, and no relationship is observed between city size and pollutant emission.

In 2016, only in Longnan was the built-up area less than 15 square kilometers. In 13 cities—Beijing, Chongqing, Guangzhou, Tianjin, Shanghai, Dongguan, Shenzhen, Chengdu, Nanjing, Qingdao, Hangzhou, Changchun, and Xi’an—the built-up areas were larger than 496 square kilometers. Thus, for most cities, the expansion of city size harms pollutant emission reduction because the built-up areas in most cities are not large enough.

In summary, according to the thresholds and the Chinese city size in 2016, Chinese cities are not too large but too small. Cities are too small to effectively use pollution treatment facilities, invest sufficient funds in environmental protection, and take advantage of pollution treatment’s scale effect.

### 4.3. Regressions inside and outside an Urban Agglomeration

An urban agglomeration is the highest structural form of organization when urban development occurs rapidly. An urban agglomeration is huge, multi-core, and multi-level, formed by many cities centrally distributed in the region. An urban agglomeration is also a combination of metropolitan areas and has the function of convergence and diffusion of various production factors. Urban agglomeration has become the most dynamic and potential economic development area and the productivity distribution’s growth pole.

By 2018, China had formally formed seven major metropolitan agglomerations: Yangtze River Delta Urban Agglomeration, Beijing-Tianjin-Hebei Urban Agglomeration, Guangdong-HongKong-Macao Greater Bay Area, Chengdu-Chongqing Urban Agglomeration, Middle Reaches of Yangtze River Urban Agglomeration, Central Plains Urban Agglomeration, and Guanzhong Plain Urban Agglomeration.

Compared with small cities, urban agglomerations have different characteristics of their environmental pollution and pollutant emission reduction. When industrialization enters the middle and late phases, population and industry constantly shift to a single-core urban agglomeration centered on a large city or multi-core urban agglomeration centered on multiple large cities, resulting in the agglomeration of population and industry.

Spatial agglomeration may have two impacts on environmental pollution. On the one hand, urban agglomeration strengthens the economies of scale effect. In an urban agglomeration, due to the clustering of the population, economy, and various treatment facilities, the efficiency of environmental infrastructure utilization will be improved greatly with increased efficiency of centralized treatment of pollution and a decrease of the average cost for pollution control. From this perspective, an urban agglomeration is more conducive to sustainable development. On the other hand, although urban agglomerations have advantages in controlling pollution due to economies of scale, environmental problems persist and are caused by a high-density population and industrial agglomeration, including water pollution caused by urban agglomeration and the superposition effect, the heat island effect of urban agglomeration, and the solid waste problem of urban agglomeration [27].

To examine the different relationship between city size and environmental pollution in cities in an urban agglomeration and cities not in an urban agglomeration, we split the sample into two groups. One group contains cities in urban agglomeration, and the other group contains cities not in urban agglomeration. Taking industrial dust discharge as the dependent variable and city size as the independent variable measured by the total urban population, GDP, and the built-up area, the regression is repeated for the two sample groups, respectively.

The regression results are presented in Table 9, the regressions for cities in an urban agglomeration are shown in Column (1), (2), and (3), and the regressions for cities not in an urban agglomeration are presented in Column (4), (5), and (6). In Table 9, the estimated coefficients of *SIZE* in cities in an urban agglomeration are all significantly positive, and the estimated coefficients of *SIZE*^2^ in cities in an urban agglomeration are all significantly negative, suggesting that the inverted U-shaped relationship between city size and environmental pollution is valid for cities in an urban agglomeration. By contrast, for the regression of cities not in an urban agglomeration, the estimated coefficients of *SIZE*^2^ are not significant in all columns. The estimated coefficients of SIZE are significant in only Column (6). Thus, the inverted U-shaped relationship between city size and environmental pollution is not valid for cities not in an urban agglomeration.

The different regression results indicate that when a city is in an urban agglomeration, with the expansion of the city size, it is possible to reach the inflection point of the inverted U-shaped curve because of the cooperation and synergy among the cities in the urban agglomeration. However, if a city is not in an urban agglomeration, the inverted U-shaped relationship between city size and environmental pollution is no longer significant. Therefore, for cities in an urban agglomeration, environmental pollution can be reduced by expanding the city size through the coordinated development of an urban agglomeration. However, for cities not in an urban agglomeration, the relationship between city size and environmental pollution is not prominent.

### 4.4. Further Regressions

The environmental pollution in a city is not only from production, but also from living. However, the cities’ environmental pollution data is from “China City Statistical Yearbook”, which is published by the National Bureau of Statistics in China. This yearbook discloses data on the discharge of pollutants such as industrial wastewater and industrial waste gas in Chinese cities at prefecture level and above but has no data on living pollution. We collected the PM_2.5_ average annual concentration data of sample cities, which is monitored by the Ministry of Ecology and Environment of the People’s Republic of China, and checked the regression results using this data. The results shown in Table 10 are robust.

## 5. Discussion

In 2020, the Chinese government proposed that “China aims to peak its carbon dioxide emissions by 2030 and achieve carbon neutrality by 2060” (named “double carbon target”) at the general debate of the 75th United Nations General Assembly. To achieve this goal, more attention should be paid to the relationship between city size and environmental pollution under the “double carbon target” according to the above conclusions.

First of all, we need a comprehensive assessment of urban environmental infrastructure and pollution. Due to the improvement of environmental infrastructure construction and the strengthening of environmental investment, some large cities have formed a system of urban environmental governance, and the pollutant emission and GDP in these cities are not commensurate, such as Xi’an in Shanxi Province, Fuzhou in Fujian Province, and Changchun in Jilin Province. However, although the population and GDP are small in some small cities, environmental pollution is huge, such as Liaoyang in Liaoning Province, Tongling in Anhui Province, and Jiujiang in Jiangxi Province, and this finding is particularly noteworthy. Thus, for some cities, environmental infrastructure should be strengthened, while for others, urban production and living activities could be further expanded to make full use of existing environmental infrastructure.

Secondly, we should have a scientific understanding of the “big city disease”. The environmental problems in some large cities are not due to the large scale of the city, but to the incomplete construction of the corresponding environmental infrastructure. Cities are too small to make effective use of pollution treatment facilities, make sufficient investments in environmental protection, and use the scale effect of pollution treatment. For most of the large cities in China, the city size is not too large but too small.

Finally, for cities in an urban agglomeration, environmental pollution can be reduced by expanding city size through the coordinated development of urban agglomeration. However, for cities not in an urban agglomeration, the relationship between city size and environmental pollution is not apparent.

Despite our valuable conclusions, this paper has several limitations. First, because of the limitations of the data, we could not obtain more data before 2003. Thus, we could not analyze the dynamic relationship between city size and pollutant emissions over a long period. Second, environmental pollution in cities is caused by production and living. However, we did not carry out intensive analysis into the relationship between city size and environmental pollution caused by living because of the missing data, which would be necessary to understand the mechanism between city size and environmental pollution, especially for large cities.

## 6. Conclusions

As complex environments comprising people and economic activities, cities constantly gather where goods and services are produced and consumed [1]. With the continuous development of the economy and society, cities are expanding at an alarming rate, and this phenomenon is particularly prominent in developing countries. With the rapid urban development, many factors, such as industry, population, and capital, are constantly gathering in cities, and environmental pollution problems caused by agglomeration occur. Urban diseases such as traffic, smog, and inefficiency are emerging in large cities. A critical practical question that requires an urgent answer is whether a city is too big or too small.

With a balanced panel data of 285 Chinese cities from 2003 to 2016, the relationship between city size in an urban agglomeration and environmental pollution was assessed in this research. The empirical findings and policy suggestions are as follows:

(1) From the spatial distribution of population, the economy, and pollutant emissions of Chinese cities, we observe three asymmetries in urban development.

The first asymmetry refers to the asymmetry between population and GDP. Some cities have a large population, but the GDP is not high, reflecting low efficiencies, such as Fuyang in Anhui, Zhumadian in Henan, and Lvliang in Shanxi. Some cities have a high economic development level. However, their population is small, indicating a large population expansion space in these cities, such as Dongguan in Guangdong, Dongying in Shandong, and Zhenjiang in Jiangsu.

The second asymmetry refers to the asymmetry of water pollution and air pollution. From the spatial part of pollutants in Chinese cities, water pollution is mainly distributed in the eastern coastal areas and the Yangtze River Economic Belt. At the same time, air pollution is mainly distributed in North China and Northwest China. Therefore, a differentiation policy should be adopted for different pollutants and different regions.

The third asymmetry refers to the asymmetry between city size and environmental pollution. Large cities may have better environmental performance, while small cities may have worse environmental quality.

(2) The relationship between city size and environmental pollution is not linear but an inverted U-shape. When the city’s population size gradually expands, the pollution emissions first increase and then decrease after the inflection point. Whether GDP or built-up area is used as a measure of city size, the inverted U-shape remains. When the city size enlarges to the inflection point, the environmental pollution decreases following the expansion of city size.

(3) The inflection points of the population are 9.42 million when the environmental pollution is represented by industrial wastewater discharge, 2.25 million when the environmental pollution is represented by industrial SO_2_ discharge, and 6.09 million when the environmental pollution is represented by industrial dust discharge. According to the three different inflection points represented by the different pollutant emissions, the samples were divided into four groups. The first group has 62 cities with a population smaller than 2.25 million; the second group has 158 cities with a population smaller than 6.09 million and larger than 2.25 million; the third group samples are 47 cities with a population smaller than 9.42 million and larger than 6.09 million; the fourth group samples are 18 cities with a population larger than 9.42 million. Only for the fourth group, with the expansion of city size, the gas and water pollutant emissions decrease.

Most existing studies believe that city size and environmental pollution have a linear relationship and ascribe environmental problems in big cities to the oversize of cities, which ignores the inflection point between urban scale and environmental pollution. From this point of view, the conclusion of this study theoretically supplements and expands the existing research on urbanization and environmental pollution.

## Figures and Tables

**Table 1:** Summary statistics of the key variables.

Variable	Unit	Obs	Mean	Std Dev	Min	Max
*POLLU*						
*wastewater*	thousand tons	3990	74.396	95.795	0.070	912.600
*so2*	thousand tons	3990	58.475	58.754	0.002	683.162
*dust*	thousand tons	3990	32.976	120.421	0.034	5168.812
*SIZE*						
*population*	ten thousand	3990	431.85	305.23	16.37	3392
*gdp*	billion RMB	3990	1496.699	2303.817	31.773	28,178.650
*area*	square kilometers	3990	113.134	162.450	5	1420
*IND*	%	3990	48.646	11.05	9	90.97
*FDI*	billion dollars	3990	6.375	16.504	0	308.256
*TECH*	%	3990	2.52	5.04	0	36.81
*ENERGY*	billion-kilowatt hour	3990	72.650	127.435	0.225	1486.020
*INVEST*	billion RMB	3990	924.874	1267.146	16.567	17,245.760

**Table 2:** Regression results (population size).

	Population Size		
	Industrial Wastewater	Industrial SO_2_	Industrial Dust
	(1)	(2)	(3)
*SIZE*	2.205 **	2.718 **	4.963 ***
	(2.30)	(2.37)	(3.38)
*SIZE* ^2^	−0.161 *	−0.251 **	−0.387 ***
	(−1.89)	(−2.46)	(−2.97)
*IND*	0.409 ***	1.106 ***	0.119
	(6.35)	(14.34)	(1.21)
*FDI*	0.011	0.012	−0.038 ***
	(1.46)	(1.38)	(−3.43)
*TECH*	−0.019 ***	−0.052 ***	−0.016 *
	(−3.34)	(−7.69)	(−1.80)
*ENERGY*	0.108 ***	0.109 ***	0.082 **
	(4.82)	(4.06)	(2.37)
*INVEST*	−0.116 ***	−0.189	0.013
	(−8.09)	(−11.05)	(0.57)
Adjust R^2^	0.870	0.858	0.715
F statistics	15.94	58.93	7.21
Model	FE	FE	FE
Observation	3990	3990	3990

Note: T statistics are in parentheses; *** *p* < 0.01, ** *p* < 0.05, and * *p* < 0.1.

**Table 3:** Regression results (GDP and built-up area).

	Gdp			Built-Up Area		
	Industrial Wastewater	Industrial SO_2_	Industrial Dust	Industrial Wastewater	Industrial SO_2_	Industrial Dust
	(1)	(2)	(3)	(4)	(5)	(6)
*SIZE*	0.490 **	2.707 ***	1.424 ***	−0.028	1.088 ***	0.984 ***
	(1.99)	(9.32)	(3.79)	(−0.20)	(6.49)	(4.57)
*SIZE* ^2^	−0.012 *	−0.086 ***	−0.033 ***	−0.008	−0.124 ***	−0.090 ***
	(−1.69)	(−9.90)	(−2.93)	(−0.55)	(−6.80)	(−3.82)
*IND*	0.373 ***	0.856 ***	0.058	0.371 ***	0.991 ***	0.046
	(5.51)	(10.72)	(0.57)	(5.61)	(12.62)	(0.46)
*FDI*	0.010	0.015	−0.040 ***	0.009	0.014 *	−0.035 ***
	(1.39)	(1.70)	(−3.60)	(1.26)	(1.67)	(−3.18)
*TECH*	−0.017 ***	−0.037 ***	−0.012	−0.016 ***	−0.046 ***	−0.011
	(−2.85)	(−5.47)	(−1.39)	(−2.80)	(−6.74)	(−1.24)
*ENERGY*	0.099 ***	0.115 ***	0.022	0.131 ***	0.103 ***	0.066 *
	(4.10)	(4.05)	(0.60)	(5.75)	(3.79)	(1.89)
*INVEST*	−0.161 ***	−0.182 ***	−0.190 ***	−0.088 ***	−0.201 ***	−0.020
	(−5.37)	(−5.15)	(−4.17)	(−5.79)	(−11.12)	(−0.84)
Adjust R^2^	0.870	0.830	0.717	0.870	0.827	0.716
F statistics	15.06	74.40	9.23	15.55	65.38	8.53
Model	FE	FE	FE	FE	FE	FE
Observation	3990	3990	3990	3990	3990	3990

Note: T statistics are in parentheses; *** *p* < 0.01, ** *p* < 0.05, and * *p* < 0.1.

**Table 4:** Inflection points calculated from the regressions.

City Size	Unit	Industrial Wastewater	Industrial SO_2_	Industrial Dust
Population	Million	9.42	2.25	6.09
GDP	Billion Yuan RMB	7362	68	23,461
Built-up area	Square kilometer	Inverted U-shape is not invalid	80.40	236.75

**Table 5:** Threshold regression results (population).

Threshold Point	Industrial Wastewater	Industrial SO_2_	Industrial Dust
$\gamma_{1}$	1,320,000	570,000	570,000
$\gamma_{2}$	1,840,000	1,040,000	3,300,000
$\gamma_{3}$	2,160,000	3,340,000	11,470,000
*SIZE* ($thr\leq \gamma_{1}$)	0.258	0.167	−0.001
	(1.56)	(0.76)	(−0.00)
*SIZE* ($\gamma_{1}<thr\leq \gamma_{2}$)	0.159	−0.702 ***	0.381 *
	(1.03)	(−3.80)	(1.75)
*SIZE* ($\gamma_{2}<thr\leq \gamma_{3}$)	0.231	−0.544 ***	0.504 **
	(1.57)	(−3.09)	(2.37)
*SIZE* ($thr>\gamma_{3}$)	0.033 **	−0.469 ***	0.399 *
	(2.70)	(−2.72)	(1.87)
*IND*	0.370 ***	1.083 ***	0.175 *
	(5.98)	(14.75)	(1.90)
*FDI*	0.011 *	0.0101	−0.029 ***
	(1.68)	(1.26)	(−2.89)
*TECH*	−0.016 ***	−0.042 ***	−0.015 *
	(−2.79)	(−6.03)	(−1.69)
*ENERGY*	0.131 ***	0.143 ***	0.130 ***
	(6.15)	(5.61)	(4.06)
*INVEST*	−0.099 ***	−0.141 ***	0.031
	(−7.45)	(−8.86)	(1.53)
Adjust R^2^	0.864	0.819	0.718
F statistics	12.89	45.79	22.97
Method	FE	FE	FE
Observation	3990	3990	3990

Note: T statistics are in parentheses; *** *p* < 0.01, ** *p* < 0.05, and * *p* < 0.1.

**Table 6:** Threshold regression results (GDP).

Threshold Point	Industrial Wastewater	Industrial SO_2_	Industrial Dust
$\gamma_{1}$	544,068	12,355	41,641
$\gamma_{2}$		850,713	800,370
*SIZE* ($thr\leq \gamma_{1}$)	0.013	−0.034 ***	0.013 ***
	(0.41)	(−8.86)	(4.94)
*SIZE* ($\gamma_{1}<thr\leq \gamma_{2}$)	−0.111 ***	−0.048	0.459 ***
	(−6.60)	(−0.89)	(6.67)
*SIZE* ($\gamma_{2}<thr$)		−0.026 ***	−0.024 ***
		(−5.82)	(−4.49)
*IND*	0.372 ***	1.001 ***	0.176 *
	(6.07)	(13.51)	(1.85)
*FDI*	0.010	0.009	−0.034 ***
	(1.55)	(1.15)	(−3.20)
*TECH*	−0.017 **	−0.036 ***	−0.017 *
	(−2.89)	(−5.18)	(−1.91)
*ENERGY*	0.139 ***	0.132 ***	0.062 *
	(6.59)	(4.83)	(1.79)
*INVEST*	−0.090 ***	−0.129 ***	−0.155 ***
	(−7.00)	(−3.82)	(−3.64)
Adjust R^2^	0.865	0.820	0.253
F statistics	20.23	55.23	499.23
Method	FE	FE	FE
Observation	3990	3990	3990

Note: T statistics are in parentheses; *** *p* < 0.01, ** *p* < 0.05, and * *p* < 0.1.

**Table 7:** Threshold regression results (built-up area).

Threshold Point	Industrial Wastewater	Industrial SO_2_	Industrial Dust
$\gamma_{1}$	500	15	15
$\gamma_{2}$		830	496
*SIZE* ($thr\leq \gamma_{1}$)	0.067 ***	−0.344 ***	−0.190 ***
	(4.75)	(−9.97)	(−4.36)
*SIZE* ($\gamma_{1}<thr\leq \gamma_{2}$)	−0.124 ***	−0.035	0.249 ***
	(−3.63)	(−0.87)	(4.88)
*SIZE* ($\gamma_{2}<thr$)		−0.093 ***	−0.110 ***
		(−5.26)	(−5.17)
*IND*	0.337 ***	1.007 ***	0.081
	(5.48)	(13.68)	(0.87)
*FDI*	0.0109	0.012	−0.028 ***
	(1.62)	(1.49)	(−2.72)
*TECH*	−0.014 **	−0.039 ***	−0.011
	(−2.48)	(−5.65)	(−1.29)
*ENERGY*	0.151 ***	0.136 ***	0.100 ***
	(6.96)	(5.29)	(3.08)
*INVEST*	−0.077 ***	−0.146 ***	0.009
	(−5.41)	(−8.62)	(0.42)
Adjust R^2^	0.865	0.820	0.715
F statistics	17.22	56.53	21.27
Method	FE	FE	FE
Observation	3990	3990	3990

Note: T statistics are in parentheses; *** *p* < 0.01, ** *p* < 0.05.

**Table 8:** Results summary.

	Industrial Wastewater	Industrial SO_2_	Industrial Dust
Population	[0, 2,160,000] ^#^ (2,160,000, ∞) ^+^	[0, 570,000] ^#^ (570,000, ∞) ^-^	[0, 570,000] ^#^ (570,000, ∞) ^+^
GDP	[0, 544,068] ^#^ (544,068, ∞) ^-^	[0, 12,355] or (850,713, ∞) ^-^ (12,355, 850,713] ^#^	[0, 800,370] ^+^ (800,370, ∞) ^-^
Built-up area	[0, 500] ^+^ (500, ∞) ^-^	[0, 15] or (830, ∞) ^-^ (15, 830] ^#^	[0, 15] or (496, ∞) ^-^ (15, 496] ^+^

Note: ^#^ no relationship between city size and pollutant emission, ^+^ positive relationship between city size and pollutant emission, ^-^ negative relationship between city size and pollutant emission.

**Table 9:** Results summary (cities in and not in an urban agglomeration).

	Cities in an Urban Agglomeration			Cities Not in an Urban Agglomeration		
	Population	GDP	Built-Up Area	Population	GDP	Built-Up Area
	(1)	(2)	(3)	(4)	(5)	(6)
*SIZE*	6.982 ***	2.832 ***	0.974 ***	0.964	−0.059	0.678 **
	(3.62)	(4.61)	(3.09)	(0.41)	(−0.10)	(2.11)
*SIZE* ^2^	−0.532 ***	−0.068 ***	−0.091 ***	−0.092	0.010	−0.048
	(−3.14)	(−3.87)	(−2.81)	(−0.41)	(0.55)	(−1.28)
*IND*	−0.034	−0.362 **	−0.222	0.243 *	0.291 **	0.262 **
	(−0.21)	(−2.02)	(−1.37)	(1.90)	(2.23)	(2.00)
*FDI*	−0.077 ***	−0.087 ***	−0.076 ***	−0.024 *	−0.024 *	−0.021
	(−3.31)	(3.73)	(−3.24)	(−1.91)	(−1.91)	(−1.61)
*TECH*	−0.006	−0.005	−0.002	−0.020	−0.023 *	−0.018
	(−0.49)	(−0.44)	(−0.13)	(−1.52)	(−1.73)	(−1.42)
*ENERGY*	0.117 **	0.022	0.116 **	0.070	0.028	0.037
	(2.06)	(0.35)	(2.00)	(1.59)	(0.61)	(0.85)
*INVEST*	−0.057 *	−0.394 ***	−0.070	0.111 ***	−0.034	0.052
	(−1.72)	(−5.55)	(−1.99)	(3.47)	(−0.55)	(1.62)
Adjust R^2^	0.685	0.687	0.682	0.739	0.740	0.741
F statistics	7.14	8.50	4.86	8.29	9.35	10.43
Model	FE	FE	FE	FE	FE	FE
Observation	2030	2030	2030	1960	1960	1960

Note: T statistics are in parentheses; *** *p* < 0.01, ** *p* < 0.05, and * *p* < 0.1.

**Table 10:** Regression results (PM_2.5_).

	Population Size	GDP	Built-Up Area
	(1)	(2)	(3)
*SIZE*	0.051 ***	0.002 ***	−0.003
	(30.48)	(3.26)	(−0.49)
*SIZE* ^2^	−0.000 ***	−0.000 ***	−0.000 ***
	(−19.05)	(−3.42)	(−2.66)
*IND*	0.495 ***	0.323 ***	0.302 ***
	(20.25)	(11.97)	(11.04)
*FDI*	−0.027	−0.122 ***	−0.149 ***
	(−0.83)	(−3.23)	(−4.18)
*TECH*	0.292 **	0.163	0.201
	(2.01)	(1.00)	(1.26)
*ENERGY*	−0.009 **	−0.014 **	0.005
	(−2.55)	(−2.51)	(1.12)
*INVEST*	−0.000	0.004 ***	0.006 ***
	(−0.06)	(6.18)	(11.95)
Adjust R^2^	0.266	0.090	0.092
F statistics	205.58	56.11	57.25
Model	FE	FE	FE
Observation	3990	3990	3990

Note: T statistics are in parentheses; *** *p* < 0.01, ** *p* < 0.05.

## Data Availability

The data can be made available upon request.
